# Expression of the inhibitory Ly49E receptor is not critically involved in the immune response against cutaneous, pulmonary or liver tumours

**DOI:** 10.1038/srep30564

**Published:** 2016-07-29

**Authors:** Jessica Filtjens, Jiri Keirsse, Els Van Ammel, Sylvie Taveirne, Aline Van Acker, Tessa Kerre, Tom Taghon, Bart Vandekerckhove, Jean Plum, Jo A. Van Ginderachter, Georges Leclercq

**Affiliations:** 1 Laboratory of Experimental Immunology, Ghent University, Ghent, Belgium; 2 Myeloid Cell Immunology Lab, VIB Inflammation Research Center, Ghent, Belgium; 3 Lab of Cellular and Molecular Immunology, Vrije Universiteit Brussel, Brussels, Belgium

## Abstract

Natural killer (NK) lymphocytes are part of the innate immune system and are important in immune protection against tumourigenesis. NK cells display a broad repertoire of activating and inhibitory cell surface receptors that regulate NK cell activity. The Ly49 family of NK receptors is composed of several members that recognize major histocompatibility complex class I (MHC-I) or MHC-I-related molecules. Ly49E is a unique inhibitory member, being triggered by the non-MHC-I-related protein urokinase plasminogen activator (uPA) in contrast to the known MHC-I-triggering of the other inhibitory Ly49 receptors. Ly49E also has an uncommon expression pattern on NK cells, including high expression on liver DX5^−^ NK cells. Furthermore, Ly49E is the only Ly49 member expressed by epidermal γδ T cells. As γδ T cells and/or NK cells have been shown to be involved in the regulation of cutaneous, pulmonary and liver malignancies, and as uPA is involved in tumourigenesis, we investigated the role of the inhibitory Ly49E receptor in the anti-tumour immune response. We demonstrate that, although Ly49E is highly expressed on epidermal γδ T cells and liver NK cells, this receptor does not play a major role in the control of skin tumour formation or in lung and liver tumour development.

T cells present in thymus and peripheral lymphoid organs have a large repertoire of T cell receptors (TCRs) composed of either αβ or γδ heterodimers. In mice, γδ T cells represent only a small fraction of lymphocytes circulating the peripheral blood and lymphoid organs. Instead, several γδ T cells subsets belong to the intra-epithelial lymphocytes (IELs) and they are the main T cell population found in epithelial tissues, such as skin, intestine and reproductive tract^1^,^2^. Mouse γδ IELs have a restricted TCR diversity and develop in different waves during foetal ontogeny, giving rise to γδ T cells bearing TCRs composed of different variable (V) γ and δ regions^2^. Vγ3 T cells (nomenclature by Garman *et al*.^1^) are the first T cells to arise in the foetal thymus^2^,^3^, where they mature upon Skint-1-mediated positive selection^2^,^4^. Thereafter, Vγ3 T cells migrate to the epidermis^2^. An important function of epidermal Vγ3 T cells is the regulation of cutaneous malignancies. Girardi *et al*. showed that TCR δ^−/−^ mice are more susceptible to develop epidermal malignancies and treatment of these mice with the carcinogen 3-methylcholanthrene (3-MCA) results in increased tumour development as compared to WT mice^5^. The precise role of γδ T cells in the protective immune response against cutaneous tumour development was further studied by Gao *et al*.^6^. They demonstrated that γδ T cells play a critical role in the protective immune response against tumour development through provision of an early source of IFN-γ, that in turn regulates the function of tumour-specific αβ T cells. This was shown by analysis of tumour formation and growth in C57BL/6 TCR δ^−/−^ compared to WT mice upon either intradermal injection of 3-MCA or subcutaneous injection of B16 melanoma cells. Both models resulted in increased tumour development in the absence of γδ T cells. Besides γδ T cells, also natural killer (NK) cells are involved in the protection against B16 melanoma-induced tumours^6^.

Contrary to subcutaneous injection, intravenous injection of B16 cells results in tumour cell colonization of the lungs^6^. *In vivo* depletion of NK cells by anti-asialo GM1 antibody or anti-NK1.1 monoclonal antibody (mAb) augments the pulmonary tumour load and also induces liver tumour nodules, which are not observed in NK-sufficient mice^6^. This clearly demonstrates a role for NK cells in the anti-B16 tumour immune response. Additionally, liver NK cells are involved in the immune protection against hepatocellular carcinoma (HCC)^7^. This is the most abundant type of primary liver cancer with an immunosuppressive microenvironment characterized by functionally impaired T and NK cells^8^,^9^. It has been shown in a murine orthotopic HCC model that stimulation of CD137, a member of the tumor necrosis factor (TNF) receptor family, with an agonistic antibody leads to complete tumour regression in 40–60% of the animals. Depletion of NK cells or T cells abrogated this anti-tumour effect, pointing them out as the main mediators therein^7^.

NK cells express a broad repertoire of inhibitory and activating cell surface receptors, which restrain and induce NK cell reactivity, respectively^10^,^11^. Inhibitory NK receptors of the Ly49 family and the inhibitory CD94/NKG2 receptors recognize classical MHC-I molecules and the nonclassical MHC-I molecule Qa-1b, respectively^10^,^12^. In contrast, the activating receptor NKG2D recognizes induced-self proteins that appear on the surface of stressed, malignant transformed or infected cells^10^,^11^. Consequently, NK cell activation occurs 1) when MHC-I molecules on transformed or infected cells are absent or reduced, eliminating the inhibitory signal (missing-self recognition), or 2) when transformed or infected cells display increased expression of stimulatory ligands, overcoming the constitutive inhibition delivered by inhibitory receptors and leading to activation (induced-self recognition).

Also Vγ3 T cells express NK receptors. The majority of the cells expresses NKG2D^13^, while CD94/NKG2 is expressed by 60% of the Vγ3 T cells^14^. Ly49 members are rarely expressed, with the exception of Ly49E that is present on 60% of foetal thymic Vγ3 T cells and 20% of epidermal Vγ3 T cells^14^. Importantly, the percentage of Ly49E-expressing epidermal Vγ3 T cells increases to 60% after TCR stimulation^15^. Ly49E is a unique member of the murine Ly49 NK receptor family with several characteristics that clearly distinguish this receptor from other Ly49 receptors. Whereas other inhibitory Ly49 receptors bind classical MHC-I ligands, this is not the case for Ly49E^16^. Ly49E, instead, is triggered by urokinase plasminogen activator (uPA), a non-MHC-I molecule^17^. uPA is a well-studied protein. It is a serine protease that cleaves inactive plasminogen to generate plasmin^18^. Plasmin also belongs to the serine proteases and has a wide range of functions both in non-pathological processes, such as tissue remodelling and wound healing, and in pathological conditions, including tumour growth and metastasis. It contributes to tumour development by its ability to cleave and activate precursor forms of certain matrix metalloproteases that then degrade many extracellular matrix components, a crucial step in cancer invasion and metastasis^19^. High uPA levels in cancer patients are associated with increased malignancies in a variety of cancer types^20^,^21^. Our hypothesis is that the Ly49E receptor, triggered by uPA produced by tumour cells, inhibits immune subpopulations involved in the anti-tumour response, thereby revealing a novel tumour immune escape mechanism.

During ontogeny, Ly49E is the first Ly49 receptor expressed by NK cells and it is the only Ly49 receptor expressed by foetal and neonatal NK cells^17^,^22^,^23^. Although Ly49E expression is low on conventional peripheral NK cells in adult mice^14^,^22^,^24^, approximately 20% of DX5^−^ liver NK cells express Ly49E in adult mice^25^. In addition, also approximately 20% of epidermal γδ T cells express Ly49E, and Ly49E expression increases to 60% upon T cell receptor-mediated stimulation^14^,^15^. As NK cells and epidermal γδ T cells are important in the control of lung/liver and cutaneous malignancies, respectively, and as both immune cell populations express the Ly49E receptor, we studied whether this NK receptor regulates the anti-tumour immune response. We therefore tested several tumour models in Ly49E KO versus WT mice. The results show that the inhibitory Ly49E receptor has no major role in the immunosurveillance of B16F10-induced lung and liver tumours, of orthotopic Hepa 1–6-induced HCC, or of 3-MCA- or B16F10-induced cutaneous tumours.

## Results

### Ly49E expression is not critically involved in immune protection against 3-MCA-induced cutaneous tumourigenesis

**Figure 1:**
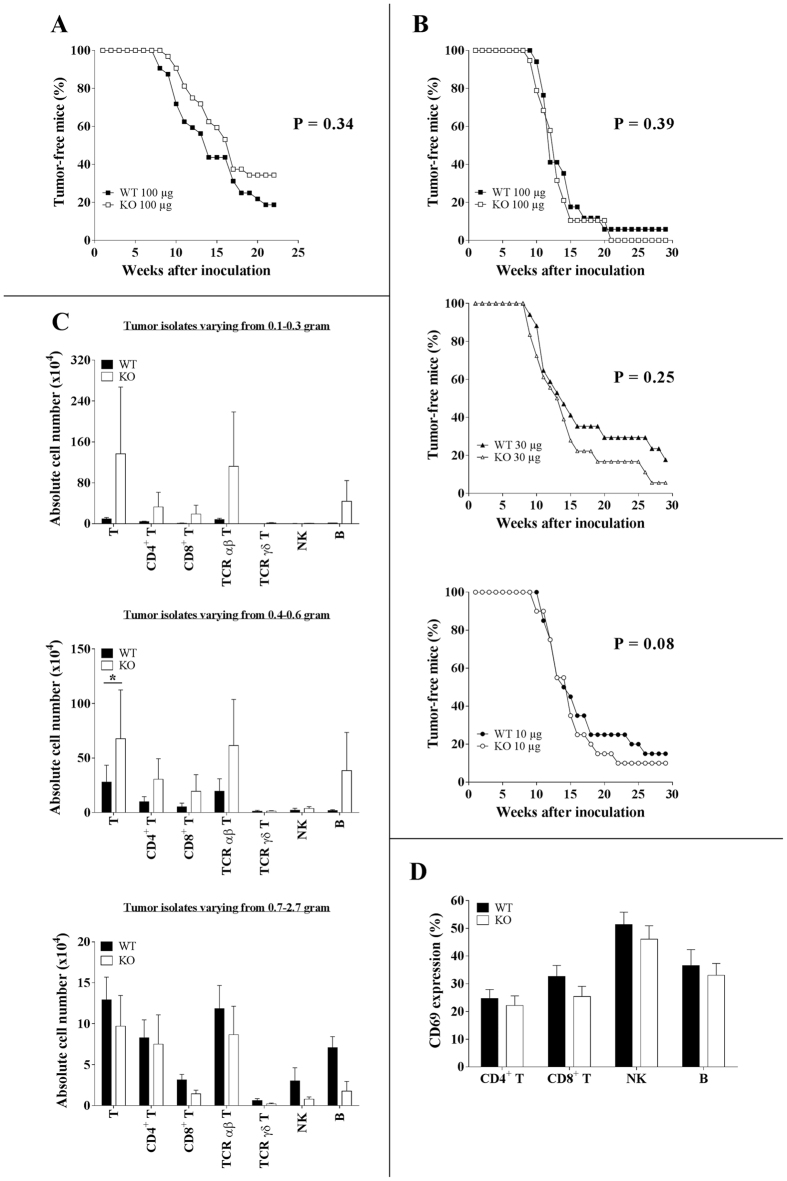
Ly49E expression is not critically involved in immune protection against 3-MCA-induced cutaneous tumourigenesis. **(A**) Sex- and age-matched C57BL/6 WT (■; n = 32) and Ly49E KO mice (□; n = 32) were intradermally injected in the flank with 100 μg 3-MCA. Tumour development was recorded weekly and is presented as the percentage of tumour-free mice. (**B**) Three groups of sex- and age-matched WT and Ly49E KO mice were injected with either 100 μg (WT (■), n = 17; Ly49E KO (□), n = 19), 30 μg (WT (▲), n = 17; Ly49E KO (Δ), n = 18) or 10 μg 3-MCA (WT (●), n = 20; Ly49E KO (○), n = 20). Tumour development was analysed. (**A**,**B**) Data presented in **A** and **B** are from two different experiments. Datasets were statistically analysed using the Log-rank Kaplan-Meier method. The P value is indicated. There were no significant differences in WT vs Ly49E KO mice. (**C,D**) Tumour-infiltrating cells were analysed in mice injected with 100 μg 3-MCA. (**C**) Data are presented as the absolute cell number (mean ± s.e.m.) of the indicated lymphocyte subpopulations in tumour isolates varying from 0.1-0.3 gram (WT (■), n = 10; Ly49E KO (□), n = 8), 0.4–0.6 gram (WT (■), n = 10; Ly49E KO (□), n = 11) and 0.7–2.7 gram (WT (■), n = 10 ; Ly49E KO (□), n = 4). (**D**) The percentage of CD69-positive cells (mean ± s.e.m.) in the indicated lymphocyte subpopulations (WT (■), n = 24; Ly49E KO (□), n = 21). (**C,D**) Datasets were statistically analysed using the non-parametric 2-tailed Mann-Whitney U-test. * P ≤ 0.05.

Lymphocytes, together with IFN-γ, are known to be important for tumour immunosurveillance^26^. The involvement of γδ T cells in inhibiting 3-MCA-induced fibrosarcoma development in C57BL/6 mice was demonstrated by Girardi *et al*.^5^ and Gao *et al*.^6^. To test the regulatory role of the inhibitory Ly49E receptor expressed by skin Vγ3 T cells in their anti-tumour response, sex- and age-matched C57BL/6 WT and Ly49E KO mice were intradermally injected in the flank with 100 μg 3-MCA, and tumour development was monitored and measured weekly. There was no significant difference in tumour incidence and onset in Ly49E KO versus WT mice (Fig. 1A). We reasoned that reducing the 3-MCA dose, and thus lowering tumour incidence and/or progression, might reveal intrinsic differences in the anti-tumour immune response of WT and Ly49E KO mice. We therefore injected both WT and Ly49E KO mice with 10 or 30 μg 3-MCA, and also repeated the 100 μg 3-MCA injection. However, also with lower MCA concentrations, there was no significant difference in tumour incidence and onset in Ly49E KO mice compared to WT mice (Fig. 1B).

Next, we compared the tumour-infiltrating lymphocyte subsets in Ly49E KO versus WT mice. Tumour cell suspensions were made and flow cytometric analysis was performed. As the tumours varied in size and as the relative numbers of tumour-infiltrating lymphocytes are different in small versus large tumours, we grouped the mice according to the tumour size: from 0.1 to 0.3, from 0.4 to 0.6, and from 0.7 to 2.7 gram tumour tissue. There was no difference in the mean tumour size in KO compared to WT mice (data not shown). Figure 1C shows the absolute number of different subsets of tumour-infiltrating immune cells comparing Ly49E KO to WT mice in these 3 groups of tumour isolates, as indicated. The largest population of tumour-infiltrating cells were T cells, that were predominant αβ T cells. γδ T cells, and in particular Vγ3 T cells, were only present in low cell numbers. Both in the 0.1–0.3 gram and 0.4–0.6 gram tumour isolates, there was a trend in higher cell numbers of tumour-infiltrating T and B cells in Ly49E KO compared to WT mice, but the variability in the KO mice was high. Only in the total tumour-infiltrating T cells in 0.4–0.6 gram tumour isolates there was a significant increased cell number in KO as compared to WT mice. We further analysed expression of the early activation marker CD69 on the different lymphocyte subsets. CD69 was expressed on part of the CD4^+^ and CD8^+^ αβ T cells, NK cells and B cells, whereas γδ T cells were almost CD69 negative (Fig. 1D and data not shown). There was no significant difference in CD69 expression in WT versus Ly49E KO mice. Cumulatively, these results indicate that Ly49E expression on epidermal Vγ3 T cells is not involved in the protection against cutaneous 3-MCA-induced tumour development.

### B16F10 and Hepa 1–6 tumour cells express uPA

In addition to 3-MCA-induced fibrosarcoma, we wanted to test other tumour models for the role of the Ly49E receptor in the cellular immune response. As indicated in the introduction, B16F10 tumour cells can either be injected subcutaneously or intravenously to give rise to skin tumours or lung and liver tumours, respectively. In the skin tumour model, increased tumour development is observed in the absence of γδ T cells, whereas also NK cells are involved^6^. NK cells are also important in the immune response in the lung and liver B16 tumour model. Additionally, liver NK cells are involved in the immune protection against hepatocellular carcinoma (HCC) induced by orthotopic injection of Hepa 1–6 tumour cells^7^.

**Figure 2:**
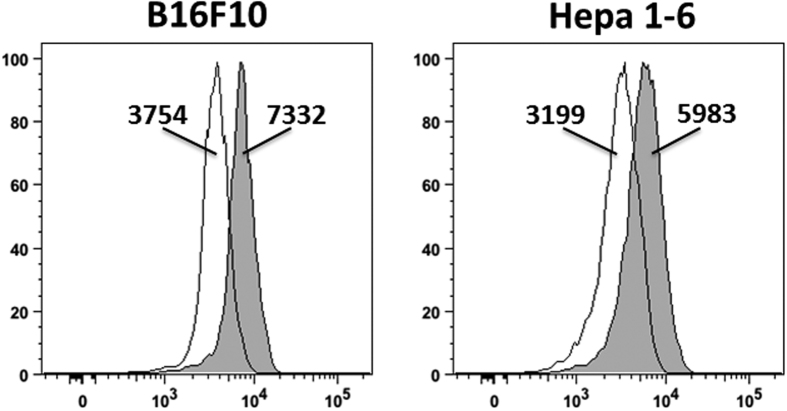
B16F10 and Hepa 1–6 tumour cells express uPA. uPA expression was analysed in B16F10 and Hepa 1–6 tumour cells by flow cytometry using biotinylated anti-uPA antibody, revealed with PE-conjugated streptavidin (filled histogram) or second step only (empty histogram). The geomean fluorescence intensity is indicated. Results shown are representative for 2 experiments.

To test whether B16F10 and Hepa 1–6 cells express uPA, we analysed these tumour cells by flow cytometry. The results show that both tumour cell lines express uPA (Fig. 2), and thus these tumour models are suitable to investigate the role of Ly49E expression in the anti-tumour immune response.

### Cutaneous B16F10 tumour development is not influenced by Ly49E expression

**Figure 3:**
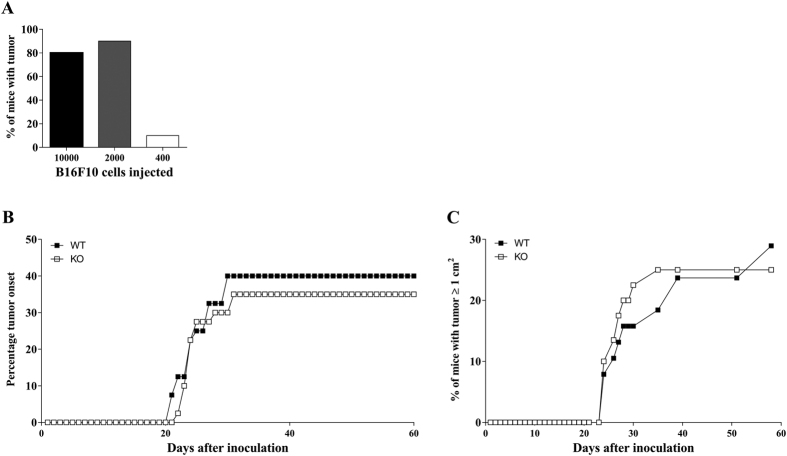
The involvement of the Ly49E receptor in cutaneous B16F10-induced tumour development. (**A**) WT mice were inoculated subcutaneously with the indicated number of B16F10 melanoma cells (5 mice with 10^4^ cells, 10 mice with 2000 cells and 10 mice with 400 cells) and tumour development was monitored. (**B**) Age- and sex-matched WT (■) (n = 40) and Ly49E KO mice (□) (n = 40) were injected subcutaneously with 1500 B16F10 melanoma cells and tumour growth was analysed. The percentage of tumour onset is shown in (**B**), the percentage of mice bearing tumours larger than 1 cm^2^ is shown in (**C**). The data shown are representative of two independent experiments. (**B,C**) Datasets were statistically analysed using the Log-rank Kaplan-Meier method. There were no significant differences in WT versus Ly49E KO mice.

To further explore the potential role of the Ly49E receptor in regulation of cutaneous tumour surveillance, we induced cutaneous tumours by subcutaneous injection of B16F10 melanoma cells. Gao *et al*.^6^ showed that γδ T cells have a protective role in this tumour model and that they are the first immune cells to be recruited to the site of injection. First, we performed a titration experiment to define the optimal number of injected B16F10 cells. WT C57BL/6 mice were injected with 10000, 2000 and 400 B16F10 cells and tumour development was monitored (Fig. 3A). On the basis of these results, we decided to inject 1500 B16F10 cells in the next experiments, as this cell number would induce tumours in a substantial part of the group, but not in all mice. WT and Ly49E KO mice were compared and tumour development was recorded on a daily basis. There were no significant differences in tumour onset kinetics and tumour incidence in WT versus Ly49E KO mice (Fig. 3B). When analysing the kinetics of appearance of tumours ≥1 cm^2^, there was also no statistically difference in WT versus Ly49E KO mice (Fig. 3C).

### Role of the Ly49E receptor in the B16F10 lung/liver tumour model

**Figure 4:**
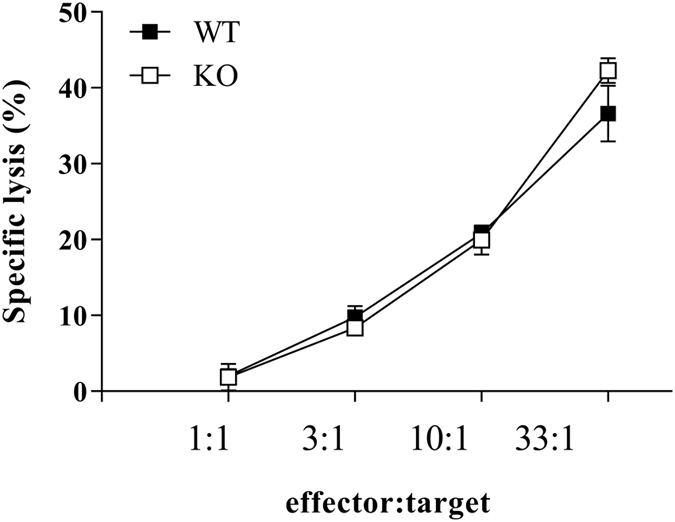
*In vitro* sensitivity of B16F10 tumour cells to liver NK cell-mediated cytotoxicity. The cytotoxic activity of liver NK cells from WT (■) and Ly49E KO mice (□) against B16F10 target cells is shown. Data shown are from one single experiment with duplicate samples and are presented as the mean ± s.e.m. specific lysis.

In addition to the involvement of γδ T cells in the protection against B16 melanoma-induced lung and liver tumours, it was shown that also NK cells play a critical role in this model. Takeda *et al*.^27^ demonstrated that the number of lung metastatic nodules following intravenous injection of B16 cells increases after NK cell depletion by anti-asialo GM1 antibody or anti-NK1.1 mAb. Importantly, they also showed that NK cell depletion induces liver tumour nodules, which are not detected in the presence of NK cells. Because Ly49E is highly expressed on liver NK cells^25^, we investigated the role of Ly49E in pulmonary and liver tumour development. First, we examined the sensitivity of B16F10 cells to NK cell-mediated cytotoxic activity *in vitro*. Freshly isolated liver mononuclear cells, depleted for CD3^+^ T cells, from WT and Ly49E KO mice were used in a ^51^Cr release assay. B16F10 tumour cells were efficiently killed by liver NK cells, with similar cytotoxic activity in liver NK cells from WT versus Ly49E KO mice (Fig. 4).

**Figure 5:**
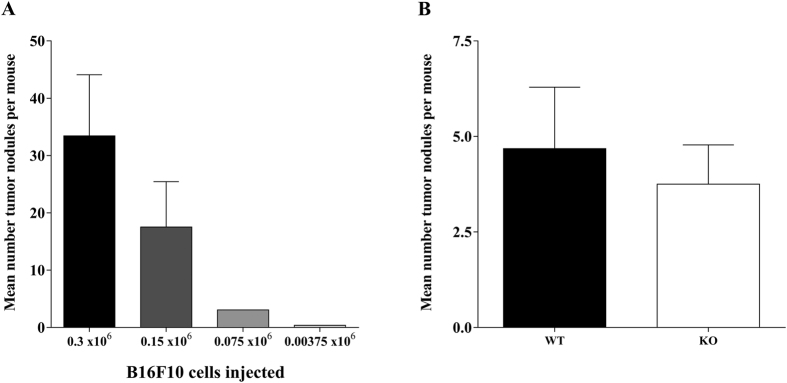
Ly49E involvement in pulmonary tumour development. (**A**) Number of lung metastatic nodules 14 days following intravenous inoculation of the indicated number of B16F10 cells into WT mice (mean ± s.e.m.) (0.3 × 10^6^ B16F10 cells: n = 6; 0.15 × 10^6^ and 0.075 × 10^6^ B16F10 cells: n = 7; and 0.0375 × 10^6^ B16F10 cells: n = 9). (**B**) Number of lung metastatic nodules 14 days following intravenous inoculation of 0.1 × 10^6^ B16 cells into WT mice (■) (n = 19) and Ly49E KO mice (□) (n = 20). Results are presented as mean ± s.e.m. There was no significant difference in the number of lung nodules in WT vs Ly49E KO mice (non-parametric 2-tailed Mann-Whitney U-test). (**A,B**) Data presented in A and B are from two different experiments.

Next, we performed an *in vivo* titration experiment in which WT mice were injected intravenously with 0.3 × 10^6^, 0.15 × 10^6^, 0.075 × 10^6^ or 0.037 × 10^6^ B16F10 cells. The injected tumour cell number correlated well with the number of induced lung tumour nodules at day 14 (Fig. 5A). Based on these results, we decided to inject WT and Ly49E KO mice with 0.1 × 10^6^ B16F10 cells in the next experiment. There was no significant difference in the number of lung nodules at day 14 after inoculation in Ly49E KO mice compared to WT mice (Fig. 5B). Liver nodules were never detected (data not shown).

### Hepa 1–6 HCC development is similar in WT and Ly49E KO mice

**Figure 6:**
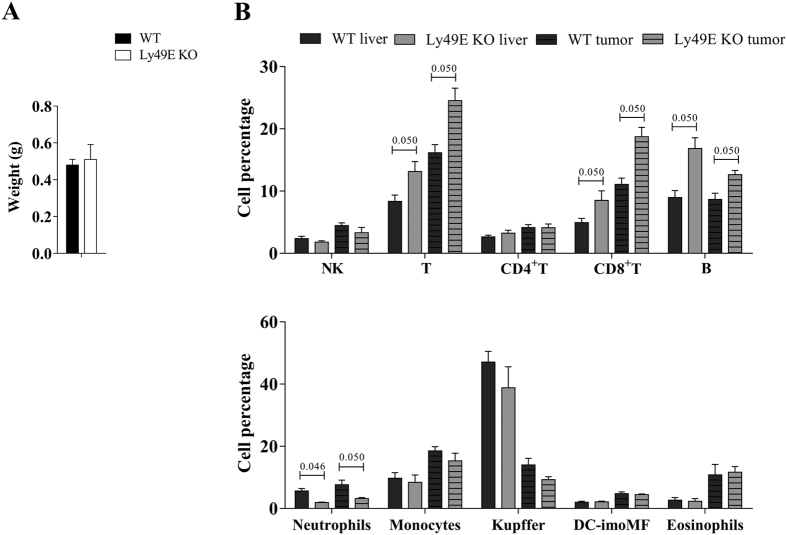
Hepa 1–6-induced HCC development is unaltered in Ly49E KO mice. (**A**) Weight of the left large liver lobe 7 days following orthotopic inoculation of Hepa1–6 cells. (**B**) Leukocyte subpopulations in tumour-free liver tissue (liver) and in tumour-infiltrating cells (tumour) were analysed and compared in WT versus Ly49E KO mice, as indicated. Data are presented as the percentage of the indicated leukocyte subpopulations relative to the total CD45^+^ leukocyte population. Data shown are from one experiment (WT: n = 3; Ly49E KO: n = 3) and are presented as mean ± s.e.m. Statistical analysis was performed using the non-parametric 2-tailed Mann-Whitney test. When P ≤ 0.05, the P value is indicated. Only significant statistical differences between WT and Ly49E KO are shown.

It is known that NK cells play an important role in the anti-tumour response against orthotopic Hepa 1–6-induced HCC^7^. Together with our knowledge that liver NK cells express high Ly49E levels^25^, we compared Hepa 1–6-induced HCC in WT versus Ly49E KO mice. The tumour size was determined at day 7 after inoculation. The left large liver lobe, in which Hepa1–6 cells were injected, was isolated and weighed. There was no significant difference in tumour growth in WT versus Ly49E KO mice based on the liver lobe weight (Fig. 6A). We also analysed leukocyte subpopulations in tumour-free liver tissue and in tumour-infiltrating cells, and compared WT to Ly49E KO mice. Different lymphoid and myeloid subpopulations were addressed. The gating strategy during flow cytometric analysis is indicated in Supplementary Fig. S1. Overall, comparing tumour-infiltrating cells to tumour-free liver tissue, the percentage of CD8 T cells, monocytes and eosinophils was higher, and that of Kuppfer cells was lower. There were intrinsic differences in the percentage of CD8^+^ T cells, B cells and neutrophils in tumour-free liver tissue of WT compared to Ly49E KO mice, and these differences were equally present in the tumour-infiltrating cells of these mice (Fig. 6B).

## Discussion

Extensive research has been performed to define the role of the immune system in cancer control. It has become clear that both the adaptive and innate immune system are involved in anti-tumour responses. The role of the adaptive immune system in tumour surveillance has been well studied, whereas the role of the innate immune system has received less attention. Innate NK cells are well equipped to kill tumour cells. NK cells express both activating and inhibitory receptors that can function with some independence, making it possible to differentially target cells that have altered their ligand expression^10^,^11^. The regulatory role of NK receptors in the anti-tumour immune response has been most extensively studied for NKG2D, a dominant activating receptor present on almost all NK cells and on part of γδ T cells. Ligands for this receptor are poorly expressed by healthy cells, but are often upregulated by tumour cells^28^. Regarding the role of NKG2D in tumour immunosurveillance, Guerra *et al*.^29^ showed a threefold increase in early-arising prostate tumours in NKG2D-deficient as compared to WT mice. Moreover, analysis of NKG2D ligand expression in tumours from both strains showed that early tumours of WT mice lack NKG2D ligands, while these ligands are present in NKG2D-deficient mice. This can be explained by immunoediting, a process where immune responses result in the selection of variant tumour cells that have lost expression of specific molecules or antigens that are targeted by immune effector cells^30^. However, tumour cells can also evade immunosurveillance by secreting soluble NKG2D ligands. This results in reduction of cell membrane NKG2D expression, leading to a decrease in NKG2D-mediated immune responses^31^. However, Deng *et al*.^32^ recently showed that in mice a shed form of MULT1, a high-affinity NKG2D ligand, causes NK cell activation and tumour rejection. They also demonstrated that soluble MULT-1 functions, at least in part, by competitively reversing a global desensitization of NK cells imposed by engagement of membrane NKG2D ligands on tumour-associated cells, such as myeloid cells. These results show that regulation of the anti-tumour immune response by NK receptors is very complex.

Also in skin cancer, NKG2D has a role in the immunosurveillance. Girardi *et al*.^5^ demonstrated increased expression of transcripts for NKG2D ligands on skin tumours induced by chemical carcinogens. Blocking the T cell receptor of Vγ3 T cells results in decreased *in vitro* killing capacity towards the tumour cells. This effect is amplified by adding soluble Rae-1ε, which is one of the NKG2D ligands expressed on tumour cells, or by antibody-mediated blocking of the NKG2D receptor on Vγ3 T cells. Thus, epidermal Vγ3 T cells kill skin tumours upon TCR- and NKG2D-mediated activation. Also Guerra *et al*.^29^ studied the role of the NKG2D receptor in skin tumour surveillance. In this study, NKG2D-deficient and WT mice were compared for 3-MCA-induced tumour formation. However, in contrast to the results of Girardi *et al*.^5^, Guerra *et al*.^29^ could not find a significant role for the NKG2D receptor in epidermal tumour immunosurveillance as there was no change in 3-MCA tumour formation in NKG2D-deficient mice versus WT mice.

In addition to the activating NKG2D receptor, there is also evidence that inhibitory NK receptors play an important role in immunosurveillance. Recently, Tu *et al*.^33^ investigated the importance of Ly49 receptors in this process. Therefore, they developed a mutant mouse strain in which expression levels of all Ly49 receptors are downregulated. Subcutaneous injection of B16F10 melanoma cells results in a significant increase in tumour incidence and tumour size in Ly49-mutant mice as compared to WT mice. Induction of tumours by subcutaneous 3-MCA injection results in earlier sarcoma onset and a significantly increased relative tumour growth in Ly49-mutant mice versus WT mice. Furthermore, the number of pulmonary metastases upon intravenous injection of B16F10 cells is higher in Ly49-mutant mice as compared to WT controls. These data demonstrate the importance of Ly49 receptors in NK cell-mediated tumour immunosurveillance. Tu *et al*.^33^ argue that education of NK cells, in which interaction between inhibitory Ly49 receptors and self MHC-I molecules during development results in the acquisition of NK cell functionality, is reduced in Ly49-mutant mice.

In this study, we investigated the role of the Ly49E receptor in tumour surveillance. At the time we demonstrated that uPA triggers the inhibitory Ly49E receptor^17^, the function of uPA in tumour biology was already extensively shown. uPA is a plasminogen activator that converts plasminogen to plasmin, which in turn degrades fibrin and other extracellular matrix constituents^34^,^35^. As uPA binds to the urokinase receptor uPAR, it plays an important role in localized cell-associated proteolysis, including tissue remodelling and cell migration^35^,^36^. A range of important functions for uPA in tumour invasion and metastasis have been revealed, most of which are linked with the binding of uPA to uPAR. As uPAR facilitates pro-uPA activation, it increases the generation of plasmin and thus proteolysis of extracellular matrix components and the basement membrane^37^,^38^. On the other hand, uPA-activated uPAR influences cell migration itself, as it associates to transmembrane integrins and hence induces several signal transduction cascades^39^. By these 2 mechanisms, cancer cells can move across the extracellular matrix and the basement membrane in order to reach blood vessels and other organs. As a consequence, uPA is a prognostic marker in a large variety of cancers, where high levels of uPA in tumour tissue predict poor outcome^40^. Our hypothesis was that the inhibitory Ly49E receptor expressed by NK cells and innate-like T cells is triggered by uPA produced by tumour cells. This would reveal a novel tumour immune escape mechanism. To experimentally test our hypothesis, we evaluated several tumour models and we compared WT to Ly49E KO mice. Ly49E KO mice and WT mice were intradermally injected with 3-MCA, or subcutaneously injected with B16F10 tumour cells. Although epidermal γδ T cells have an important role in tumour development^5^,^6^ and Ly49 receptors are involved in cutaneous immunosurveillance^33^, the absence of Ly49E did not influence tumour onset or progression. Also, although there was a trend in increased tumour-infiltrating immune cells in Ly49E KO mice as compared to WT mice, these differences were not statistically significant. This might indicate that although not critical for the tumour development outcome, immune cell number alterations in KO suggest that Ly49E may play an inhibitory role on the cellular response to some extent. Ly49 receptors are also important in preventing pulmonary B16F10 metastases^33^ and NK cells are involved in the protection against lung and liver tumours formed by B16F10 melanoma cells^6^. Therefore, we also studied pulmonary and liver metastases after intravenous injection of B16F10 melanoma cells in Ly49E KO and WT mice. Liver tumour nodules were never detected and there was no significant difference in the number of lung metastases in Ly49E KO mice compared to WT mice. Finally, we tested an orthotopic HCC model, in which NK cells were shown to be anti-tumourigenic^7^. However, we did not observe significant differences in tumour growth between WT and Ly49E KO mice, indicating that Ly49E does not play a major role in the immune response against HCC.

In conclusion, although Ly49E is expressed on skin γδ T cells, which are involved in cutaneous tumour surveillance, the absence of the Ly49E receptor does not have an effect on 3-MCA-, nor on B16F10-induced skin tumour development. Also B16F10-induced pulmonary and liver tumours, as well as Hepa1–6 cell-induced HCC are unaltered in Ly49E KO mice.

## Methods

### Animals and tumour induction

WT and Ly49E KO mice (C57BL/6 background)^25^ were bred and housed in our SPF animal facility in individually ventilated cages. Both male and female mice were used in the experiments, and mice were age-matched between WT and Ly49E KO mice. All animal experimentation was performed after approval and according to the guidelines of the Ethical Committee for Experimental Animals at the Faculty of Medicine and Health Sciences of Ghent University, Ghent, Belgium (ethics committee protocol number ECD12/07) and of the ‘Ethische commissie voor dierproeven’ at the Vrije Universiteit Brussel, Brussels, Belgium (ethics committee protocol number 13-220-4).

3-MCA-induced fibrosarcomas were achieved by intradermal injection of the carcinogenic substance. 3-MCA (Sigma-Aldrich, St. Louis, MO, USA) was dissolved in corn oil. Sex- and age-matched groups of mice were shaved and injected intradermally with 10, 30, or 100 μg 3-MCA in the right flank. Mice were monitored weekly for development of fibrosarcomas and weighed. Alternatively, B16F10 melanoma cells (kindly provided by Dr. M. Smyth, QIMR Berghover Medical Research Institute, Herston, Queensland, Australia) were injected subcutaneously in the flank. Skin tumour growth was monitored daily for 59 days. B16F10 tumour cells were also injected intravenously to induce lung tumours. Mice were weighed on a daily basis, sacrificed at day 14 after inoculation and lung tumours were counted. Finally, HCC was induced by injection of Hepa 1–6 cells (ATCC). Under anaesthesia, 2 × 10^6^ Hepa 1–6 cells were injected in the left large liver lobe of ten- to twelve-week old male WT and Ly49E KO mice. At day 7 after inoculation, mice were sacrificed and the left large liver lobe was isolated and weighed.

### Antibodies

mAb used for labelling were anti-NK1.1 (phycoerythrin-cyanine-7 (PE/Cy7)-conjugated, clone PK136), anti-CD49b (allophycocyanin (APC)-conjugated, clone DX5), anti-CD3 (pacific blue-conjugated, clone 145-2C11), anti-CD69 (biotin-conjugated, clone H1.2F3), anti-γδ-TCR (fluorescein (FITC)-conjugated, clone GL3), anti-CD45 (PE-conjugated, clone 30F11), anti-CD19 (biotin-conjugated, clone 1D3), anti-Ly6C (pacific blue-conjugated, clone AL-21), anti-B220 (V500-conjugated, clone RA3-6B2) and anti-CD8α (APC-conjugated, clone Lyt-2) (all from BD Biosciences, San Jose, CA, USA). Anti-Ly6G (FITC-conjugated, clone RB6-8C5), anti-CD11b (PE/Cy7)-conjugated, clone M1/70), anti-NK11 (PE-conjugated, clone PK136) and anti-Siglec F (PE-conjugated, clone 1RNM44N) (all from eBiosciences, Vienna, Austria). Anti-Ly49E/C (biotin-conjugated, clone 4D12)^22^ and anti-Ly49E/F (biotin-/FITC-conjugated, clone CM4, kindly provided by Dr. C. G. Brooks, University of Newcastle upon Tyne, Newcastle, U.K.)^23^. Anti-CD4 (peridinin chlorophyll protein Cyanine dye 5.5 (PercpCy5.5)-conjugated clone L3T4), anti-CD45 (APC-Cy7-conjugated, clone 30-F11) and anti-MHC-II (PercpCy5.5-conjugated, clone M5/114.15.2) (all from Biolegend, ImTec Diagnostics, N.V., Antwerp, Belgium). Anti-Vγ3 (DyLight-536; provided by J. Allison, University of Texas MD Anderson Cancer Center, Houston, Texas, USA), anti-αβ-TCR (biotin-/FITC-conjugated, clone H57-597) and anti-F4/80 (APC-conjugated, clone Cl:A3-1, Serotec). Rabbit anti-mouse uPA (IgG fraction, biotinylated, Molecdular Innovations, Novi, MI, USA). Biotinylated mAbs were detected with streptavidin (APC-eFluorTM780-conjugated or PE-conjugated, eBiosciences and BD Biosciences, respectively). Before staining, the FcR was blocked with anti-FcRII/III mAb (unconjugated, clone 2.4G2, kindly provided by Dr. J. Unkeless, New York, NY, USA). Live and dead cells were discriminated by using propidium iodide (Invitrogen Corporation). Samples were measured using an LSR II flow cytometer and analysed with FACSDiva 6.1.2 software (BD Biosciences).

### Flow cytometric analysis of tumour-infiltrating lymphocytes and tumour cells

As mentioned, mice were monitored weekly (3-MCA) or daily (B16F10) and tumour size was measured. When 3-MCA fibrosarcomas reached 1 cm^2^, tumours were resected. The tumour tissue was placed in 5 ml digestion solution (1 mg/ml collagenase IV, 1 mg/ml dispase II and 0.2 mg/ml DNAse I (Roche; Vilvoorde, Belgium) in RPMI-1640 medium) and minced with small scissors. After shaking for 20 min at 37 °C, cold RPMI-1640 medium supplemented with 10% foetal calf serum (FCS) (referred to as ‘RPMI 10%’) was added to stop the reaction. The suspension was passed through a 70 μm filter and washed two times with cold RMPI 10%. Tumour-infiltrated leukocytes were isolated by 37.5%/80% Percoll density centrifugation. Cells were counted with trypan blue to exclude dead cells and cultured overnight in RPMI complete (this refers to RPMI 1640 medium supplemented with 10% FCS, 100 U/ml penicillin, 100 μg/ml streptomycin, 2 mM glutamine, and 50 μM β-mercaptoethanol; all from Invitrogen) to re-express receptors that were removed during digestion. Thereafter, cells were collected and cell surface-stained.

Mice injected with Hepa 1–6 cells were sacrificed with CO_2_ at day 7 after inoculation. The livers were perfused *in vivo* via the portal vein with 10 ml saline and cut into small pieces using scissors. The suspension was transferred into a GentleMACS^TM^ C-tube (Miltenyi Biotec; Leiden, the Netherlands) with 5 ml liver digestion medium (120 U/ml Collagenase Type III (Worthington Biochemical Corporation; Lakewood, USA) and 10 U/ml of DNase I (Roche; Vilvoorde, Belgium) diluted in HBSS) and homogenized using the liver protocol of the GentleMACS^TM^ Dissociator. Finally, 5 ml of blocking medium (HBSS with 2% (v/v) heat-inactivated FCS and 5 mM EDTA (Thermo Scientific, Life Technologies Europe B.V.; Gent, Belgium) was added to stop digestion and samples were passed through a 70-μm sterile nylon. The GentleMACS^TM^ C-tube was washed with 20 ml blocking buffer and used to rinse the filter. Samples were centrifuged at 450 g at 4 °C for 8 min, the supernatant was discarded and red blood cells were lysed using ACK lysis buffer. Finally, the lysis was neutralized by adding 25 ml blocking medium, followed by centrifugation. Cells were counted with trypan blue to exclude dead cells and used for flow cytometry.

For analysis of uPA expression by B16F10 and Hepa 1–6 tumour cells, cells were permeabilized using Cytofix/Cytoperm reagent (BD Biosciences, San Jose, CA, USA) and intracellularly stained with biotinylated anti-uPA, revealed with PE-conjugated streptavidin.

### *In vitro* assessment of NK cell cytotoxicity

Liver NK cell cytotoxicity against B16F10 cells was assessed in a standard ^51^Cr-release assay. Briefly, 10^6^ target cells were labelled with 100 μCi ^51^Cr (Perkin Elmer, Waltham, MA, USA) at 37 °C, 5% CO_2_, for 75 min. Target cells were washed three times with complete RPMI. Liver cells from 20-day-old mice were used as effector cells. Preparation of liver cell suspensions was performed as described previously^41^. Total liver cells were T cell-depleted by labelling with biotinylated anti-CD3 mAb, followed by depletion with streptavidin magnetic beads (MACS; Miltenyi Biotec, Leiden, The Netherlands). The percentage of NK cells (CD3^−^NK1.1^+^) was determined by flow cytometry and this was used to calculate the effector:target (E:T) ratio. Various E:T ratios were plated in duplicate in a V-bottomed, 96-well microtiter plate in a final volume of 100 μl/well. Cells were incubated at 37 °C, 5% CO_2_, for 4 h. Supernatant was harvested, OptiPhaseSupermix (Wallac, Turku, Finland) was added, and ^51^Cr was counted with a 1450 MicroBeta Plus liquid scintillation counter (Perkin Elmer). Percent specific lysis was calculated as follows: 100 × [(experimental release-spontaneous release)/(total release-spontaneous release)].

### Statistical analysis

Data were statistically evaluated using PASW statistics 21 software (SPSS Inc., Chicago, IL, USA) or Graphpad Prism 5 (Graphpad Software, California, USA). Datasets were analysed using the Log-rank Kaplan-Meier method or the non-parametric 2-tailed Mann-Whitney U-test. A p-value ≤ 0.05 was considered statistically significant.

## Additional Information

**How to cite this article**: Filtjens, J. *et al*. Expression of the inhibitory Ly49E receptor is not critically involved in the immune response against cutaneous, pulmonary or liver tumours. *Sci. Rep*. **6**, 30564; doi: 10.1038/srep30564 (2016).

## Supplementary Material

Supplementary Information

## References

[b1] Garman R. D., Doherty P. J. & Raulet D. H. Diversity, rearrangement, and expression of murine T cell gamma genes. Cell 45, 733–742 (1986). 3486721 10.1016/0092-8674(86)90787-7

[b2] Xiong N. & Raulet D. H. Development and selection of gammadelta T cells. Immunol. Rev. 215, 15–31 (2007). 17291276 10.1111/j.1600-065X.2006.00478.x

[b3] Weber-Arden J., Wilbert O. M., Kabelitz D. & Arden B. V delta repertoire during thymic ontogeny suggests three novel waves of gamma delta TCR expression. J. Immunol. 164, 1002–1012 (2000). 10623850 10.4049/jimmunol.164.2.1002

[b4] Boyden L. M. . Skint1, the prototype of a newly identified immunoglobulin superfamily gene cluster, positively selects epidermal gammadelta T cells. Nat. Genet. 40, 656–662 (2008). 18408721 10.1038/ng.108 PMC4167720

[b5] Girardi M. . Regulation of cutaneous malignancy by gammadelta T cells. Science 294, 605–609 (2001). 11567106 10.1126/science.1063916

[b6] Gao Y. . Gamma delta T cells provide an early source of interferon gamma in tumor immunity. J. Exp. Med. 198, 433–442 (2003). 12900519 10.1084/jem.20030584 PMC2194096

[b7] Gauttier V. . Agonistic anti-CD137 antibody treatment leads to antitumor response in mice with liver cancer. Int. J. Cancer 135, 2857–2867 (2014). 24789574 10.1002/ijc.28943

[b8] Sui Q. . NK cells are the crucial antitumor mediators when STAT3-mediated immunosuppression is blocked in hepatocellular carcinoma. J. Immunol. 193, 2016–2023 (2014). 25015826 10.4049/jimmunol.1302389

[b9] Lahmar Q. . Tissue-resident versus monocyte-derived macrophages in the tumor microenvironment. Biochim. Biophys. Acta 1865, 23–34 (2016). 26145884 10.1016/j.bbcan.2015.06.009

[b10] Lanier L. L. NK cell recognition. Annu. Rev. Immunol. 23, 225–274 (2005). 15771571 10.1146/annurev.immunol.23.021704.115526

[b11] Lanier L. L. Up on the tightrope: natural killer cell activation and inhibition. Nat. Immunol. 9, 495–502 (2008). 18425106 10.1038/ni1581 PMC2669298

[b12] Vance R. E., Kraft J. R., Altman J. D., Jensen P. E. & Raulet D. H. Mouse CD94/NKG2A is a natural killer cell receptor for the nonclassical major histocompatibility complex (MHC) class I molecule Qa-1(b). J. Exp. Med. 188, 1841–1848 (1998). 9815261 10.1084/jem.188.10.1841 PMC2212405

[b13] Whang M. I., Guerra N. & Raulet D. H. Costimulation of dendritic epidermal gammadelta T cells by a new NKG2D ligand expressed specifically in the skin. J. Immunol. 182, 4557–4564 (2009). 19342629 10.4049/jimmunol.0802439 PMC3001286

[b14] Van Beneden K. . Expression of inhibitory receptors Ly49E and CD94/NKG2 on fetal thymic and adult epidermal TCR V gamma 3 lymphocytes. J. Immunol. 168, 3295–3302 (2002). 11907085 10.4049/jimmunol.168.7.3295

[b15] Van Den Broeck T. . Differential Ly49e expression pathways in resting versus TCR-activated intraepithelial gammadelta T cells. J. Immunol. 190, 1982–1990 (2013). 23338239 10.4049/jimmunol.1200354

[b16] Hanke T. . Direct assessment of MHC class I binding by seven Ly49 inhibitory NK cell receptors. Immunity 11, 67–77 (1999). 10435580 10.1016/s1074-7613(00)80082-5

[b17] Van Den Broeck T. . Ly49E-dependent inhibition of natural killer cells by urokinase plasminogen activator. Blood 112, 5046–5051 (2008). 18784372 10.1182/blood-2008-06-164350

[b18] Irigoyen J. P., Munoz-Canoves P., Montero L., Koziczak M. & Nagamine Y. The plasminogen activator system: biology and regulation. Cell. Mol. Life Sci. 56, 104–132 (1999). 11213252 10.1007/PL00000615 PMC11146966

[b19] Kwaan H. C. & McMahon B. The role of plasminogen-plasmin system in cancer. Cancer Treat. Res. 148, 43–66 (2009). 19377918 10.1007/978-0-387-79962-9_4

[b20] Henneke I. . Inhibition of urokinase activity reduces primary tumor growth and metastasis formation in a murine lung carcinoma model. Am. J. Respir. Crit. Care Med. 181, 611–619 (2010). 20056905 10.1164/rccm.200903-0342OC

[b21] Schmitt M. . Clinical impact of the plasminogen activation system in tumor invasion and metastasis: prognostic relevance and target for therapy. Thromb. Haemost. 78, 285–296 (1997). 9198168

[b22] Van Beneden K. . Expression of Ly49E and CD94/NKG2 on fetal and adult NK cells. J. Immunol. 166, 4302–4311 (2001). 11254682 10.4049/jimmunol.166.7.4302

[b23] Fraser K. P. . NK cells developing *in vitro* from fetal mouse progenitors express at least one member of the Ly49 family that is acquired in a time-dependent and stochastic manner independently of CD94 and NKG2. Eur. J. Immunol. 32, 868–878 (2002). 11870631 10.1002/1521-4141(200203)32:3<868::AID-IMMU868>3.0.CO;2-A

[b24] Stevenaert F. . Ly49E expression points toward overlapping, but distinct, natural killer (NK) cell differentiation kinetics and potential of fetal versus adult lymphoid progenitors. J. Leukoc. Biol. 73, 731–738 (2003). 12773505 10.1189/jlb.0902443

[b25] Filtjens J. . Abundant stage-dependent Ly49E expression by liver NK cells is not essential for their differentiation and function. J. Leukoc. Biol. 93, 699–711 (2013). 23475576 10.1189/jlb.0812378

[b26] Shankaran V. . IFNgamma and lymphocytes prevent primary tumour development and shape tumour immunogenicity. Nature 410, 1107–1111 (2001). 11323675 10.1038/35074122

[b27] Takeda K. . IFN-gamma production by lung NK cells is critical for the natural resistance to pulmonary metastasis of B16 melanoma in mice. J. Leukoc. Biol. 90, 777–785 (2011). 21712396 10.1189/jlb.0411208

[b28] Spear P., Wu M. R., Sentman M. L. & Sentman C. L. NKG2D ligands as therapeutic targets. Cancer Immun. 13, 8 (2013). 23833565 PMC3700746

[b29] Guerra N. . NKG2D-deficient mice are defective in tumor surveillance in models of spontaneous malignancy. Immunity 28, 571–580 (2008). 18394936 10.1016/j.immuni.2008.02.016 PMC3528789

[b30] Dunn G. P., Old L. J. & Schreiber R. D. The immunobiology of cancer immunosurveillance and immunoediting. Immunity 21, 137–148 (2004). 15308095 10.1016/j.immuni.2004.07.017

[b31] Hilpert J. . Comprehensive analysis of NKG2D ligand expression and release in leukemia: implications for NKG2D-mediated NK cell responses. J. Immunol. 189, 1360–1371 (2012). 22730533 10.4049/jimmunol.1200796

[b32] Deng W. . Antitumor immunity. A shed NKG2D ligand that promotes natural killer cell activation and tumor rejection. Science 348, 136–139 (2015). 25745066 10.1126/science.1258867 PMC4856222

[b33] Tu M. M. . Ly49 family receptors are required for cancer immunosurveillance mediated by natural killer cells. Cancer Res. 74, 3684–3694 (2014). 24802191 10.1158/0008-5472.CAN-13-3021

[b34] Robbins K. C., Summaria L., Hsieh B. & Shah R. J. The peptide chains of human plasmin. Mechanism of activation of human plasminogen to plasmin. J. Biol. Chem. 242, 2333–2342 (1967). 4226004

[b35] Mondino A. & Blasi F. uPA and uPAR in fibrinolysis, immunity and pathology. Trends Immunol. 25, 450–455 (2004). 15275645 10.1016/j.it.2004.06.004

[b36] Blasi F. & Carmeliet P. uPAR: a versatile signalling orchestrator. Nat. Rev. Mol. Cell. Biol. 3, 932–943 (2002). 12461559 10.1038/nrm977

[b37] Cohen R. L. . Effects of urokinase receptor occupancy on plasmin generation and proteolysis of basement membrane by human tumor cells. Blood 78, 479–487 (1991). 1648983

[b38] Stahl A. & Mueller B. M. Binding of urokinase to its receptor promotes migration and invasion of human melanoma cells *in vitro*. Cancer Res. 54, 3066–3071 (1994). 8187097

[b39] Yebra M. . Requirement of receptor-bound urokinase-type plasminogen activator for integrin alphavbeta5-directed cell migration. J. Biol. Chem. 271, 29393–29399 (1996). 8910604 10.1074/jbc.271.46.29393

[b40] Duffy M. J., Maguire T. M., McDermott E. W. & O’Higgins N. Urokinase plasminogen activator: a prognostic marker in multiple types of cancer. J. Surg. Oncol. 71, 130–135 (1999). 10389872 10.1002/(sici)1096-9098(199906)71:2<130::aid-jso14>3.0.co;2-9

[b41] Filtjens J. . Contribution of the Ly49E natural killer receptor in the immune response to Plasmodium berghei infection and control of hepatic parasite development. PLoS One 9, e87463 (2014). 24498110 10.1371/journal.pone.0087463 PMC3907506

