# Supplementary figures and images for: Expression of the inhibitory Ly49E receptor is not critically involved in the immune response against cutaneous, pulmonary or liver tumours
Source: Sci Rep. 2016 Jul 29;6:30564. doi: 10.1038/srep30564 (PMC4965774; doi:10.1038/srep30564)

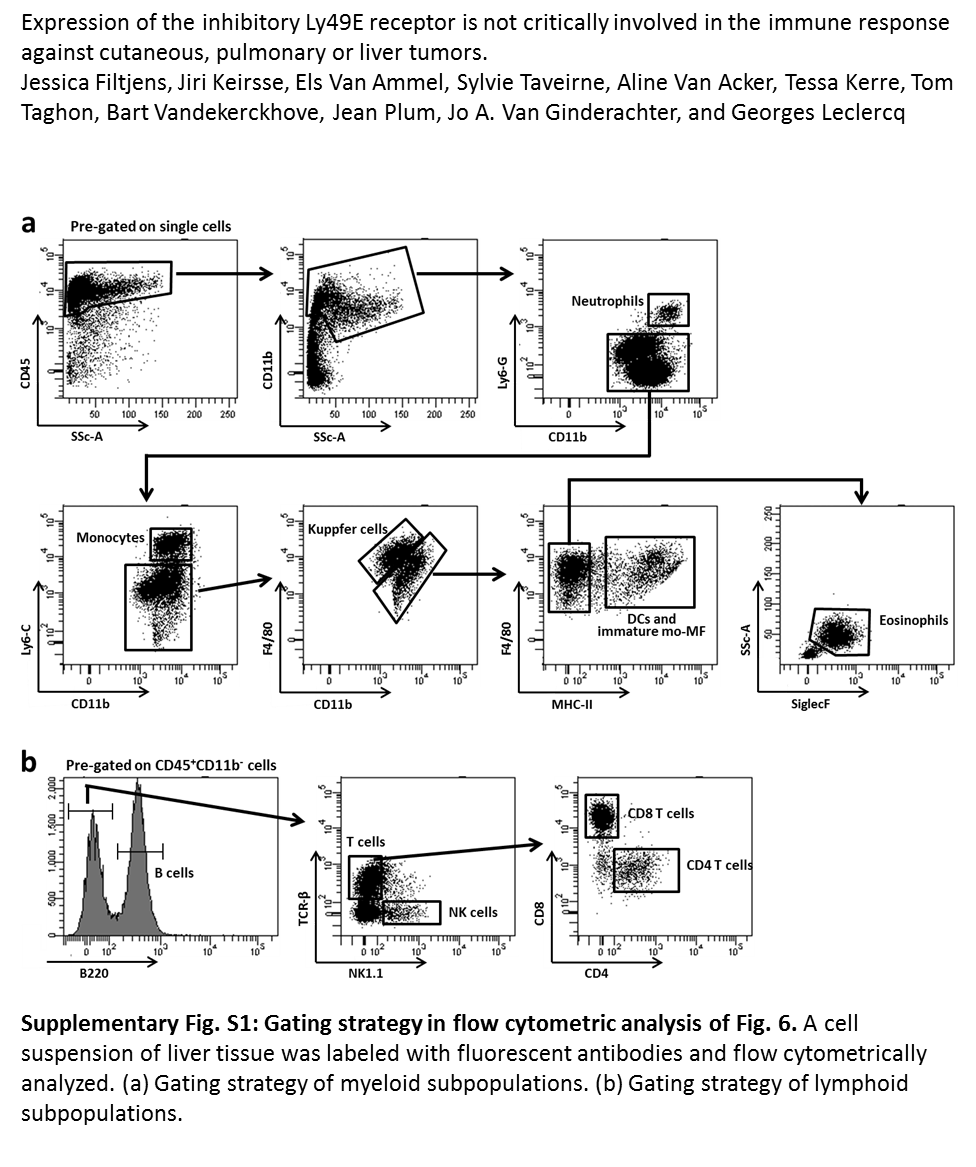

Supplement: Supplementary Information [file srep30564-s1.doc]
